# Impact of COVID‐19 on the Prevalence and Drug Resistance of *Haemophilus influenzae* in Shandong Province: A Multicenter Retrospective Study

**DOI:** 10.1002/hsr2.71459

**Published:** 2025-11-09

**Authors:** Yanmeng Sun, Mengyuan Wang, Zheng Li, Sijin Man, Shifu Wang

**Affiliations:** ^1^ Department of Clinical Microbiology Jinan Children's Hospital (Children's Hospital Affiliated to Shandong University) Jinan Shandong China; ^2^ Shandong Provincial Clinical Research Center for Children's Health and Disease Jinan Shandong China; ^3^ Department of Clinical laboratory Tengzhou Central People's Hospital Tengzhou Shandong China

**Keywords:** adult, children, China, COVID‐19, *Haemophilus influenzae*, resistance rate

## Abstract

**Background and Aims:**

The COVID‐19 pandemic has profoundly impacted global public health, particularly the epidemiology and antimicrobial resistance patterns of pathogens. This study aimed to assess the influence of COVID‐19 on the isolation rates of *Haemophilus influenzae* in Shandong Province, providing data support for empirical precision treatment in clinical practice and offering insights for future pandemic response strategies.

**Methods:**

A retrospective analysis was conducted on laboratory results and clinical records of *H. influenzae* reported by the Shandong Pediatric Antimicrobial Resistance Surveillance System from 2017 to 2023. A total of 39,043 isolates were included to analyze epidemiological trends and resistance patterns.

**Results:**

The isolation rate of *H. influenzae* significantly declined during the COVID‐19 period, reaching its lowest point in 2020, followed by a rebound in 2023. Male patients accounted for a higher proportion of cases (63.4%), and infections exhibited seasonal patterns, with the peak delayed by approximately 3 months compared to the pre‐pandemic period. The resistance rates to ampicillin were 76.2%, 85%, and 89.2% before, during, and after the pandemic, respectively. The β‐lactamase positivity rate increased annually, while the detection rate of β‐lactamase‐negative ampicillin‐resistant strains declined.

**Conclusion:**

The COVID‐19 pandemic significantly altered the epidemiological characteristics and resistance patterns of *H. influenzae* in Shandong Province. Continuous and dynamic surveillance of its trends and resistance profiles is crucial for guiding clinical treatment and infection control. These findings also provide strategic insights for global preparedness against future pandemics.

## 1 Introduction

*Haemophilus influenzae* is a small, gram‐negative bacterium that commonly colonizes the nasopharynx of healthy individuals and is known for its role in various infections. While it is typically a benign resident, it can cause respiratory infections such as community‐acquired pneumonia and suppurative tonsillitis in children. It can also lead to local infections, including acute otitis media and cellulitis. In severe cases, *H. influenzae* can enter the bloodstream, potentially resulting in sepsis, suppurative meningitis, and other invasive infections, particularly in children, the elderly, and immunocompromised individuals [1, 2].

During the COVID‐19 pandemic, many countries implemented stringent containment measures to combat the virus. These include social distancing, restrictions on movement and travel, contact tracing, restrictions on gatherings, and restrictions on outdoor activity [3]. While these actions were essential for protecting public health, they also had significant effects on people's lifestyles and habits, altering the transmission dynamics and epidemiological characteristics of various pathogens [4]. Our objective is to assess the impact of the COVID‐19 pandemic on *H. influenzae* isolated in Shandong region by exploring the characteristics and epidemiological features of the isolates before, during and after the pandemic. This impact may either alter its original epidemic patterns, such as changing the seasonal distribution characteristics, infection rate and drug resistance rate, or it may not. We aim to provide data support for early empirical and precise clinical treatment.

## 2 Materials and Methods

### 2.1 Study Site

**Figure 1:**
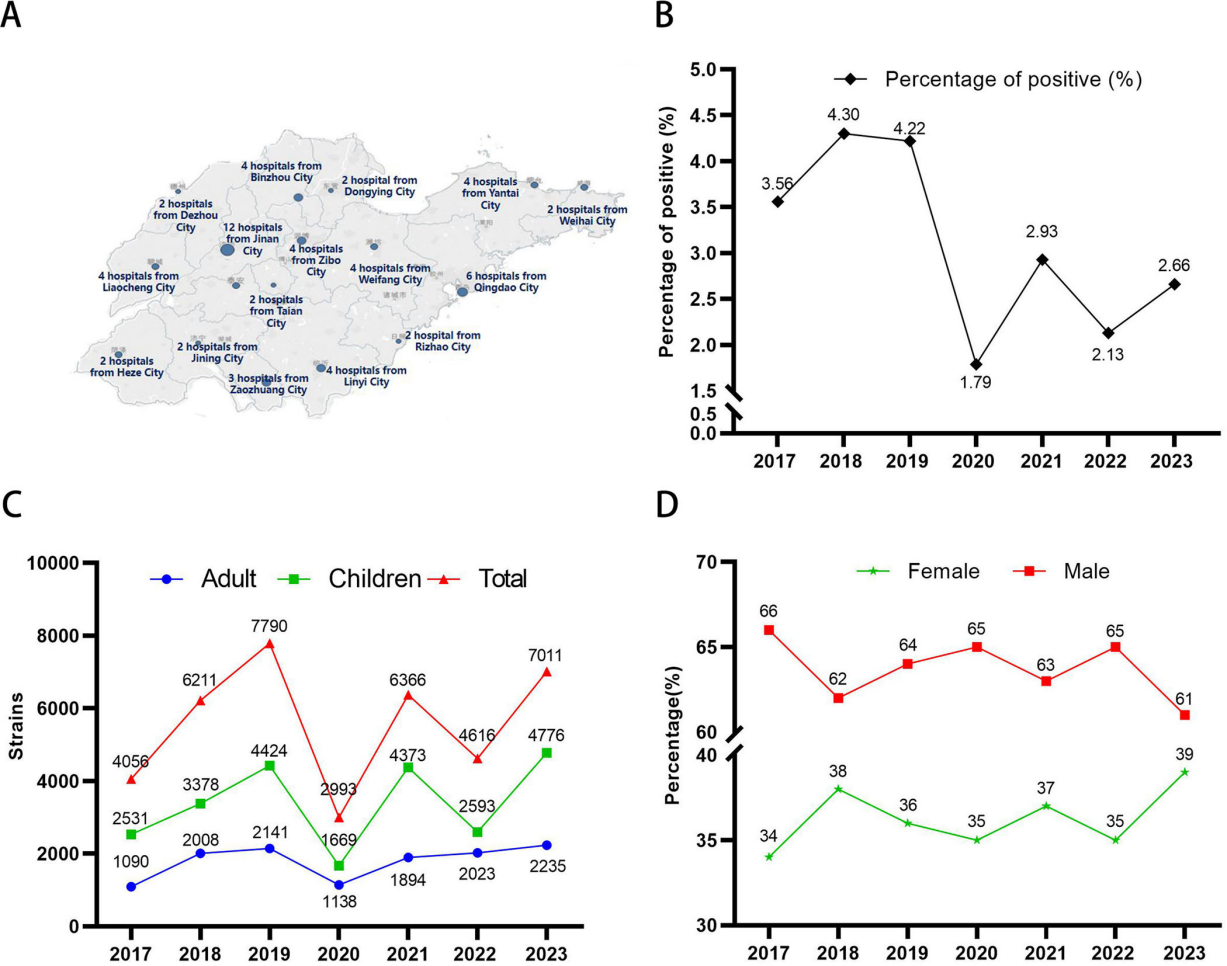
Distribution of *Haemophilus influenzae* from 2017 to 2023 in Shandong Province. (A) Map of the 59 member units of the SPARSS network. (B) The isolation rate of *H. influenzae*. (C) Number of strains isolated from adult and child. (D) Male and female proportion of *H. influenzae*.

This study was a retrospective, observational, multicenter surveillance initiative officially launched in 2017 by the Microbiology Laboratory at the Children's Hospital affiliated with Shandong University, which served as the core institution. The study included 59 participating medical institutions, consisting of 52 tertiary hospitals and 7 secondary hospitals, collectively forming the Shandong Province Pediatric Antimicrobial Resistance Surveillance System (SPARSS) net (Figure 1A). The selection of SPARSS network members was guided by principles of geographic representation and demographic distribution to ensure comprehensive coverage and high data representativeness. The network encompassed 16 major cities in Shandong Province. Within the SPARSS network, each participating hospital designated a laboratory liaison officer responsible for data collection and transmission. This arrangement ensured that the core laboratory could receive high‐quality monitoring data for the preceding year at the start of each year. Additionally, the SPARSS network maintained a commitment to open cooperation and resource sharing in scientific research, hosting annual academic conferences where member institutions engaged in detailed discussions on research advancements, technical challenges, and data interpretation, and collaboratively analyzed the previous year's monitoring data. This study was reviewed and approved by the Ethical Review Committee of Children's Hospital Affiliated to Shandong University (approval no. SDFE‐IRB/P‐2022017).

### 2.2 Ethics Statement

This study was reviewed and approved by the Ethical Review Committee of Children's Hospital Affiliated to Shandong University (approval no. SDFE‐IRB/P‐2022017).

### 2.3 Strain Source

Surveillance data on *H. influenzae* were collected from 59 member units between January 2017 and December 2023. Data from adult populations across all member units were also gathered to compare differences in pathogen profiles and drug resistance between adults and children. Duplicate isolates from the same source were excluded from the analysis. The inclusion criteria: *H. influenza*e strains isolated from sterile‐site specimens; for nonsterile‐site specimens, *H. influenzae* strains grown as pure culture or the dominant bacteria on the selective medium. The exclusion criterion: repeated isolation of *H*. *influenzae* strain from the same child. The inclusion criteria: *H. influenzae* strains isolated from sterile‐site specimens; for nonsterile‐site specimens, *H. influenzae* strains grown as pure culture or the dominant bacteria on *Haemophilus* selective medium. The exclusion criterion: repeated isolation of *H. influenzae* strain from the same child.

### 2.4 Methods

For strain identification, we utilized matrix‐assisted laser desorption/ionization‐time of flight (MALDI‐TOF) mass spectrometry, an automated microbial identification system, and the API system. Antimicrobial susceptibility testing (AST) was performed by disk diffusion method or automatic instrument method according to the unified scheme. The criteria for interpreting AST results adhered to the Clinical and Laboratory Standards Institute M100‐ED33 [5].

### 2.5 Definitions of the COVID‐19 Pandemic Periods

The periods from January 2017 to December 2019, January 2020 to December 2022, and January 2023 to December 2023 are designated as before, during and after the COVID‐19 pandemic, respectively.

### 2.6 Age Stratification Criteria

Children ≤ 14 years old, adults > 14 years old [6].

### 2.7 Data Analysis

Data analysis was performed using WHONET 5.6 software, while GraphPad Prism 8.0 software was employed for statistical analysis. The *χ²* test was used to compare drug resistance rates and strain compositions of bacteria. The *p* < 0.05 was considered statistically significant.

## 3 Results

### 3.1 Isolates

From 2017 to 2023, a total of 39,043 strains *H. influenzae* strains (from the same part of the same patient were excluded) were isolated from the member units of SPARSS network, Figure 1A is the map of the 59 member units of the SPARSS network. Before COVID‐19, the detection rate of *H. influenzae* was high, with an average of 4.03%. There was a decrease in the epidemic situation, with the largest decrease in 2020, which was the lowest in the past 7 years. In 2021, there was an increase, and in 2022, there was a decrease, with an average of 2.28%. It started to rise again after the COVID‐19 pandemic (Figure 1B). Among which 25,795 strains were in children and 13,248 strains were in adults, accounting for 66.1% and 33.9%, respectively, and most of them were children (Figure 1C). Male patients accounted for 63.4% (24,752/39,039) and female patients accounted for 36.6% (14,287/39,039), and the proportion of males was higher than that of females (Figure 1D).

### 3.2 Seasonal Distribution of *H. influenzae*

**Figure 2:**
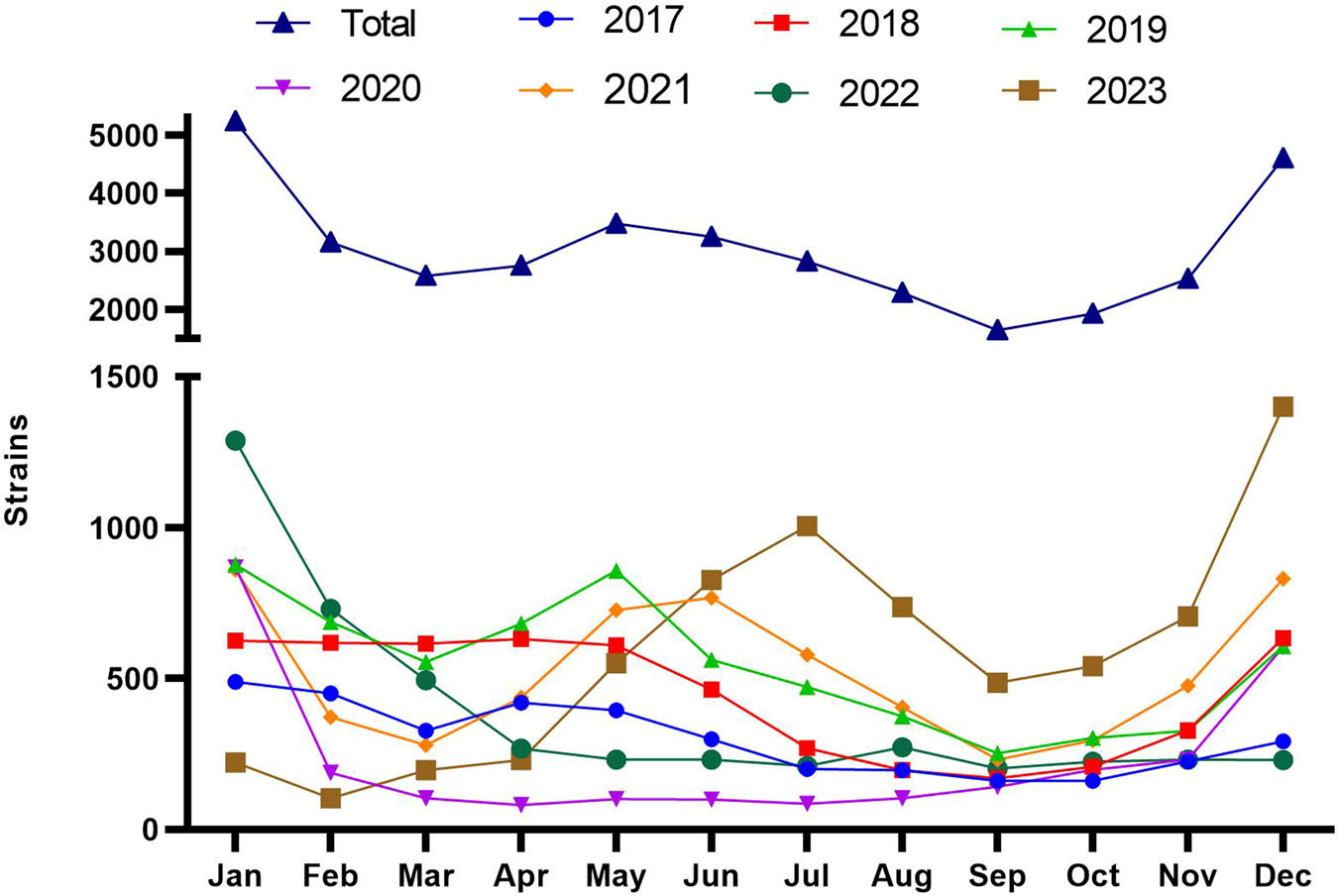
Monthly distribution of strains.

From 2017 to 2023, the seasonal distribution of *H. influenzae* is illustrated in Figure 2. Before the COVID‐19 pandemic, isolates were relatively few between August and October. The highest number of isolates was observed in January, February, May, and December, with peaks in December or January. Infections caused by *H. influenzae* exhibit certain seasonality, with higher incidence in winter and spring, and lower incidence in summer and autumn.

During the COVID‐19 pandemic, the number of isolates in 2020 was higher only in January, and remained low and steady for the following 10 months, with a resurgence beginning in December. The total number of *H. influenzae* cases detected in 2020 (354 cases) dropped to the lowest level in 4 years, which is consistent with the findings of Qing Meng [7]. In 2022, the number of isolates peaked in January, then steadily decreased until April, and remained at a low level until December.

After the COVID‐19 pandemic, in 2023, the number of isolates was low in January, began to rise gradually from April, and reached its first peak in August, which was delayed by 3 months compared to previous years. In December 2023, it reached the highest point (1401 strains) in the history of monitoring for 7 years.

### 3.3 Strain Source Distribution

A total of 96.9% (37,832 out of 39,043) of isolates were obtained from respiratory specimens, including 36,133 strains from sputum, 921 strains from throat swabs, and 778 strains from bronchoalveolar lavage fluid (BALF). Among them, there was a noticeable upward trajectory in the proportion of *H. influenzae* isolated from alveolar lavage fluid, which was observed in both children (*χ* ^2^ = 35.71, *p* < 0.001) and adults (*χ* ^2^ = 20.80, *p* < 0.001) (Table 1).

**Table 1:** The sample source of *Haemophilus influenzae* strains in children and adult from 2017 to 2023.

Group	Species	2017–2019	2020–2022	2023
**Children**		***n* = 12261**	***n* = 8758**	***n* = 4776**
	Sputum	11172 (91.1%)	8043 (91.8%)	4440 (93.0%)
	Throat	497 (4.1%)	257 (2.9%)	89 (1.9%)
	BALF	173 (1.4%)	210 (2.4%)	124 (2.6%)
	vulvar secretion	155 (1.3%)	131 (1.5%)	47 (1.0%)
	Ear	105 (0.9%)	30 (0.3%)	5 (0.1%)
	Blood	25 (0.2%)	23 (0.3%)	7 (0.1%)
	Nose	19 (0.2%)	21 (0.2%)	25 (0.5%)
	Pus	24 (0.2%)	11 (0.1%)	10 (0.2%)
	Cerebrospinal fluid	5 (0.0%)	5 (0.1%)	1 (0.0%)
	Others	86 (0.7%)	27 (0.3%)	28 (0.6%)
**Adult**		***n* = 5796**	***n* = 5217**	***n* = 2235**
	Sputum	5433 (93.7%)	4934 (94.6%)	2111 (94.5%)
	Throat	46 (0.8%)	22 (0.4%)	10 (0.4%)
	BALF	83 (1.4%)	118 (2.3%)	70 (3.1%)
	vulvar secretion	24 (0.4%)	33 (0.6%)	14 (0.6%)
	Ear	6 (0.1%)	0	1 (0.0%)
	Blood	28 (0.5%)	21 (0.4%)	10 (0.4%)
	Nose	14 (0.2%)	22 (0.4%)	1 (0.0%)
	Pus	15 (0.3%)	4 (0.1%)	2 (0.1%)
	Cerebrospinal fluid	4 (0.1%)	0	0
	Others	143 (2.5%)	63 (1.2%)	16 (0.7%)

Abbreviation: BALF, bronchoalveolar lavage fluid.

In contrast, the proportion of isolates from non‐respiratory specimens was 3.1% (1211 out of 39,043), with vulvar secretions accounting for 28.0% (404 out of 1441). Other non‐respiratory specimens included ear secretions (146), blood (114). nasal secretions (102), and pus (66). Additionally, 15 strains were identified from cerebrospinal fluid (CSF) and 364 from other specimen types (Table 1).

Examining the proportion of *H. influenzae* isolated from sputum samples of children from 2017 to 2023, we found rates of 91.1% before COVID‐19, 91.8% during the epidemic, and 93.0% after, indicating a rising trend (*χ* ^2^ = 15.41, *p* < 0.001). Conversely, the proportion of isolates from throat swabs decreased year by year, with rates of 4.1%, 2.9%, and 1.9%, respectively (*χ* ^2^ = 56.81, *p* < 0.001). On the other hand, the proportion from alveolar lavage fluid demonstrated an increasing trend, with rates of 1.4%, 2.4%, and 2.6% over the same period (*χ* ^2^ = 35.71, *p* < 0.001) (Table 1).

### 3.4 Changes in Drug Resistance Rate of *H. influenzae*

#### 3.4.1 β‐Lactamase Detection

According to β‐lactamase test results from 2017 to 2023, the detection rate of β‐lactamase in *H. influenzae* increased consistently each year, with rates of 63%, 72.1%, 76.6%, 77.1%, 84.1%, 85%, and 88% (*χ* ^2^ = 768.8, *p* < 0.001). The detection rates of β‐lactamase‐negative ampicillin‐resistant *H. influenzae* (BLNAR) were 9.7%, 7.9%, 7.3%, 6.9%, 4.8%, 4.1%, and 3.7%, respectively (*χ* ^2^ = 153.8, *p* < 0.001) (Table 2).

**Table 2:** Changing prevalence of β‐lactamase‐producing strains isolates of *Haemophilus influenzae* from 2017 to 2023.

	Year	Number	P%	Mean P%	BLNAR	BLNAR%	Mean BLNAR%
2017–2019	2017	2473	63.0	70.6	241	9.7	8.3
	2018	3000	72.1		237	7.9	
	2019	4518	76.6		328	7.3	
2020–2022	2020	1779	77.1	82.0	122	6.9	5.3
	2021	3579	84.1		172	4.8	
	2022	2262	85.0		92	4.1	
2023	2023	4768	88.0	88.0	178	3.7	3.7

*Note:* P: Prevalence of β‐lactamase‐producing strain.

The average percentage of β‐lactamase‐producing *H. influenzae* was 70.6% (average of 63%, 72.1%, and 76.6%) before the epidemic, 82% (average of 77.1%, 84.1%, and 85%) during the epidemic, and 88% after the epidemic, showing a progressive increase from before to during and after the epidemic (Table 2).

In terms of BLNAR detection rates, the average before the epidemic was 8.3% (average of 9.7%, 7.9%, and 7.3%), during the epidemic was 5.3% (average of 6.9%, 4.8%, and 4.1%), and 3.7% after the epidemic. This indicates a decreasing trend in BLNAR detection from before to during and after the epidemic (Table 2).

#### 3.4.2 Changes in Drug Resistance Rate of *H. Influenzae*

*H. influenzae* had high rates of resistance to ampicillin, cefuroxime and trimethoprim‐sulfamethoxazole and low rates of resistance to cefotaxime, meropenem, levofloxacin, and chloramphenicol (Table 3). Furthermore, the resistance rates of *H. influenzae* to ampicillin, ampicillin sulbactam and cefuroxime exhibited higher levels after the epidemic compared to those observed prior.

**Table 3:** Drug resistance rate analysis of *Haemophilus influenzae* from 2017 to 2023.

Antibiotic		Drug resistance rate (*R* %)			*p* value		
	Total	2017–2019 (*n* = 18,057)	2020–2022 (*n* = 13,975)	2023 (*n *= 7011)	2017–2019 vs. 2020–2022	2020–2022 vs. 2023	2017–2019 vs. 2023
Ampicillin	81.6	76.2	85.0	89.2	< 0.001*	< 0.001*	< 0.001*
A/S	49.9	42.9	53.0	61.9	< 0.001*	< 0.001*	< 0.001*
Cefuroxime	60.6	49	67.2	74.1	< 0.001*	< 0.001*	< 0.0001*
Cefotaxime	6.8	8.3	6.1	5.4	< 0.001*	0.26	< 0.001*
Meropenem	1.1	0	1.0	0.6	0.01*	0.02*	< 0.001*
Levofloxacin	1.3	1.8	1.0	0.8	< 0.001*	0.24	< 0.001*
Azithromycin	43.2	42.5	41.5	47.5	0.22	< 0.001*	< 0.001*
SXT	72.9	72.8	74.7	68.5	0.007*	< 0.001*	< 0.001*
Chloramphenicol	5.6	5.7	5.5	5.6	0.61	0.73	0.86

* Indicate < 0.05 A/S: Ampicillin/sulbactam SXT: Trimethoprim/sulfamethoxazole.

To compare the difference in drug resistance between β‐‐lactamase positive and negative strains, we performed a comparative analysis of drug sensitivity of the collected strains (22,379; 57.3%) with β‐lactamase test results. We find the rates of resistance to ampicillin, ampicillin‐sulbactam, azithromycin, and cefuroxime were significantly higher in beta‐lactamase‐producing *H. influenzae* than in non‐beta‐lactamase‐producing *H. influenzae* (Table 4).

**Table 4:** Drug resistance rate analysis of *Haemophilus influenzae* with β‐lactamase from 2017 to 2023.

Antibiotic	2017–2019 (*n* = 9991) (*R*%)			2020–2022 (*n* = 7620) (*R*%)			2023 (*n* = 4768) (*R*%)		
	B+ (*n* = 7179)	B− (*n* = 2812)	*p*	B+ (*n* = 6305)	B− (n = 1315)	*p*	B+ (*n* = 4197)	B− (*n* = 571)	*p*
Ampicillin	97.4	18.6	< 0.001	96.5	24.7	< 0.001	97.6	27.4	< 0.001
A/S	48.5	23.1	< 0.001	62.4	28	< 0.001	70.2	23.7	< 0.001
Cefuroxime	53.8	33.8	< 0.001	73.9	47.8	< 0.001	78.2	53.6	< 0.001
Cefotaxime	8.8	6.8	< 0.001	6.1	0	—	5.9	5.2	0.63
Meropenem	1.2	0.9	0.2	0.5	0.4	0.67	0.3	0.7	0.14
Levofloxacin	1.5	3.3	< 0.001	0.9	2.3	< 0.001	0.5	1.3	0.07
Azithromycin	52.5	7	< 0.001	42.1	8.4	< 0.001	53.3	4.8	< 0.001
SXT	76.9	60.2	< 0.001	77.8	53.7	< 0.001	70.3	52.2	< 0.001
Chloramphenicol	6.2	4.2	< 0.001	5.1	4.1	0.1414	5.2	1.1	< 0.001

Abbreviations: A/S, Ampicillin/sulbactam; B+/B−, β‐lactamase + /β‐lactamase; SXT, Trimethoprim/sulfamethoxazole.

#### 3.4.3 Changes in Drug Resistance of *H. Influenzae* in Vulvar Secretions

The amount of *H. influenzae* isolated from children's vaginal secretions is much higher than that in adults. Additionally, the drug resistance rates of *H. influenzae* in children's secretions to ampicillin, ampicillin‐sulbactam, cefuroxime and chloramphenicol exhibited a gradual increase before, during, and subsequent to the epidemic (Table 5).

**Table 5:** Drug resistance rate analysis of *Haemophilus influenzae* in vulvar secretions from 2017 to 2023.

Antibiotic		Vulvar secretion (*n *= 404) (*R*%)							
	Total	Children (*n* = 333)				Adult (*n* = 71)			
		2017–2019 (*n* = 155)	2020–2022 (*n *= 131)	2023 (*n* = 47)	*p*	2017–2019 (*n* = 24)	2020–2022 (*n* = 33)	2023 (*n* = 14)	*p*
Ampicillin	77.1	68.9	84.9	97.6	< 0.001	46.7	71.4	72.7	0.31
A/S	56.1	36.4	54.3	76.9	0.004	27.3	58.3	81.8	0.01
Cefuroxime	60.9	52.2	63.3	83.3	0.001	25	66.7	90	0.001
Cefotaxime	9.2	9.1	4.9	11.1	0.98	25	12.5	0	—
Meropenem	0.5	0	1.1	0	—	33.3	0	0	—
Levofloxacin	1.2	0.7	0	0	—	20	0	0	—
Azithromycin	37.1	35.6	37	39.1	0.73	50	55.6	20	0.35
SXT	61.2	52.7	66.1	64.5	0.09	60	75	76.9	0.31
Chloramphenicol	6.4	3.1	5.1	14.8	0.02	25	14.3	0	—

Abbreviations: A/S, Ampicillin/sulbactam; B+/B−, β‐lactamase + /β‐lactamase − ; SXT, Trimethoprim/sulfamethoxazole.

## 4 Discussion

Since the onset of pandemic, the incidence patterns and frequencies of infectious diseases such as influenza, mycoplasma, chlamydia, and respiratory syncytial virus previously following distinct epidemic rules have experienced significant alterations [8, 9, 10, 11]. *H. influenzae* is particularly susceptible to infections secondary to influenza and mycoplasma, leading to notable shifts in its detection rates and epidemic patterns before and after the COVID‐19 outbreak. This abrupt change has not only placed immense strain on healthcare systems but has also exacerbated the issue of antimicrobial resistance due to the widespread and excessive use of antibiotics, raising global concerns. Consequently, considerable efforts have been made to identify the risk factors for acquired drug resistance and to implement appropriate management, control, and preventive measures while researching the underlying mechanisms [12].

This study aims to thoroughly analyze the variations in strain sources, seasonal distribution, and antibiotic resistance of *H. influenzae* isolated from Shandong Province before and after the COVID‐19 pandemic. The goal is to elucidate the impact of COVID‐19 on the intrinsic epidemic patterns and trends of *H. influenzae*, thereby providing a robust foundation for developing more scientific and effective strategies to combat antibiotic‐resistant infections.

In this study, the proportion of *H. influenzae* isolated from children was 66.1%, which can be attributed to the immature development of the children's immune system, the limitations and abuse of antibacterial drugs [13, 14, 15]. The majority of *H. influenzae* isolates were from males (63.4%), suggesting that male is more susceptible to infection with this strain. This discrepancy may be linked to various factors, including genetic differences, immune responses variations, hormonal influences, and potentially a higher prevalence of smoking among males in Shandong Province [16, 17]. Significant differences in the isolation rates of *H. influenzae* between male and female reveal infections inequality between the sexes again.

Monitoring data revealed significant seasonal fluctuations in *H. influenzae* infections, with higher incidence rates observed in winter and spring and lower rates in summer and autumn. This pattern aligns with findings reported by Hongjiao Wang [18]. These seasonal changes may be attributed to substantial temperature fluctuations during winter and spring, which can weaken the local immune response of the airway mucosa, facilitating pathogen spread and colonization [19]. These observations indicate that the decline in *H. influenzae* isolates is closely related to the timing and stringency of COVID‐19 control measures. The subsequent increase from December 2020 to March the following year correlates with the traditional Chinese practice of celebrating the Spring Festival, during which increased population mobility and social interactions lead to a higher incidence of respiratory diseases. The peak period of *H. influenzae* in 2023 showed a backward shift of about 3 months. Therefore, it is reasonable to suppose that *H. influenzae* infections will eventually return to pre‐epidemic regularity with society returns to normal interactions [11]. Additionally, compared to previous years, the end of 2023 reached the peak of *H. influenzae* infections in December in the past 7 years. This may be because *H. influenzae* is more prone to co‐infection with mycoplasma and influenza viruses during winter and spring seasons. Thus, susceptible children should be encouraged to maintain good respiratory hygiene, wear masks, enhance their nutrient intake, improve physical fitness, and take measures to reduce the risk of *H. influenzae* infections.

The number of *H. influenzae* isolates from sputum specimens showed an increasing trend in both demographics. Following throat swabs and bronchoalveolar lavage fluid were the next most common sources. Notably, the number of *H. influenzae* isolated from bronchoalveolar lavage fluid of both children and adults before the epidemic was less than that during the epidemic. This may be attributed to the fact that COVID‐19 primarily targets the lungs, leading to lower respiratory tract infections, severe pneumonia, and acute respiratory distress syndrome in severe cases [20, 21], and bronchoscopy is one of the important means of clinical evaluation and treatment of pneumonia. The epidemic of COVID‐19 has greatly increased the frequency of bronchoscopy, thereby improving the detection rate of *H. influenzae* in bronchoalveolar lavage fluid. In non‐respiratory specimens, vulvar secretions represented the highest proportion. Among reproductive health issues in girls, vulvar vaginitis is the most common condition, with *Streptococcus pyogenes* and *H. influenzae* identified as prevalent pathogens [22, 23]. This could be related to hygiene practices among children. Previous studies have confirmed that pathogens associated with upper respiratory tract infections can be transmitted to the genital area through oral, nasal, and hand contact [24, 25]. Additionally, *Gardnerella vaginalis* is frequently found in adult vulvovaginitis cases. Given the differing pathogen compositions in childhood and adult vaginitis, accurate diagnosis of vulvovaginitis caused by *H. influenzae* in children and the judicious use of antibiotics warrant the attention of clinicians. This study suggests that *H. influenzae* causes fewer bloodstream and central nervous system infections in children and adults.

Resistance mechanisms to beta‐lactams in *H. influenzae* are including enzymatic and nonenzymatic mechanism, the most common resistant mechanism to beta‐lactams in *H. influenzae* is production of beta‐lactamases [26]. Nonenzymatic mechanism to beta‐lactams in *H. influenzae* can be mediated by modifications in cell permeability, defects in the autolytic system and in overall peptidoglycan synthesis and metabolism, or amino acid substitutions in penicillin‐binding protein 3 (PBP3) encoded by the *ftsI* gene, and alterations of the target PBPs is the most common nonenzymatic mechanism involved in beta‐lactamase resistance [26]. Historically, ampicillin has been the go‐to medication for treating *H. influenzae* infections. However, in recent years, the widespread use of antibiotics has led to a significant increase in *H. influenzae* drug resistance to ampicillin, with resistance levels becoming progressively more concerning [27]. This study observed a consistent yearly rise in the resistance rate of *H. influenzae* to ampicillin from 2017 to 2023. The primary mechanisms driving ampicillin resistance include the production of β‐lactamase and mutations in penicillin‐binding protein 3 (PBP3) [28]. The detection rate of positive β‐lactamase in *H. influenzae* exhibited an upward trajectory annually during the study period, aligning with international reports pre‐epidemic, during the epidemic, and post‐epidemic [2]. Clinical bacterial isolates collected from 11 tertiary care children's hospitals in China in 2016 to 2020 indicates that more than 60% of H. influenzae strains produced b‐lactamases [29]. *H. influenzae* from a tertiary hospital in southwest China indicates that 83.5% of the *H. influenzae* isolates were positive for β‐lactamase [28]. This trend could be attributed to the common practice of doctors prescribing antibiotics for acute upper respiratory tract infections, with *H. influenzae* being a major respiratory pathogen in both children and adults [30].

In cases of beta‐lactamase‐negative ampicillin‐resistant *H. influenzae* (BLNAR), resistance to beta‐lactams results from mutations in penicillin‐binding protein 3 (PBP3) encoded by the *ftsI* gene [31]. Interestingly, BLNAR exhibited a declining trend throughout the years surveyed, pre‐, during, and post‐epidemic, the decline in BLNAR can be attributed to the following factors. First, during the COVID‐19 pandemic, preventive measures such as mask‐wearing, social distancing, and frequent hand hygiene significantly reduced the transmission of *H. influenzae* [32]. Additionally, decreased international travel and exchanges with Japan and South Korea may have further contributed to this decline, after all, Japan and Korea have high detection rates of BLNAR [33]. Second, changes in antibiotic prescription patterns, particularly the overall reduction in antibiotic use during the pandemic, may have influenced the prevalence of drug‐resistant strains [34]. Finally, potential detection bias could have played a role, as many hospitals prioritized SARS‐CoV‐2 testing, leading to reduced surveillance of other respiratory pathogens, at the same time, some patients with mild respiratory infections were isolated at home and did not undergo bacterial culture, which may have resulted in an artificial decrease in the reported incidence of BLNAR [35]. In this study, the rate of beta‐lactamase detection in *H. influenzae* was 57.3%, which is still at a relatively low level. Failure to detect beta‐lactamase may result in underreporting of BLNAR, affecting the results of certain drugs' sensitivity, and therefore, it is necessary to strengthen the detection of beta‐lactamase in *H. influenzae*. The study also highlighted a high resistance rate of *H. influenzae* to ampicillin‐sulbactam, with a consistent upward trend observed across the years. In particular, the resistance to ampicillin‐sulbactam increased significantly (48.5% to 70.2%) in β‐lactamase‐positive strains of *H. influenzae*, which may be associated with a relatively rare BLPACR strain (β‐lactamase‐positive, amoxicillin‐clavulanate‐resistant *H. influenzae*). Due to the deletion of the breakpoint for amoxicillin‐clavulanate in the CLSI M100 32nd edition (2022), routine testing of ampicillin and sulbactam for amoxicillin‐clavulanate is no longer conducted in this region. BLPACR isolates tend to accumulate mutations in the *ftsI* gene following exposure to ampicillin. These mutations reduce the affinity of PBP3 for β‐lactam antibiotics, leading to increased resistance to cephalosporins. Therefore, it is crucial to enhance monitoring efforts for these strains. Following the Expert Recommendations on Diagnosis and Treatment of *H. influenzae* Infection in Children, it is advised to consider third‐generation cephalosporins as the primary empirical treatment, with carbapenem antibiotics like meropenem reserved for severe cases and patients allergic to cephalosporins.

The study noted a relatively high resistance rate of *H. influenzae* to azithromycin, potentially linked to its common co‐infection with mycoplasma, with azithromycin often being the preferred treatment for mycoplasma and chlamydia infections in children. Conversely, the resistance rate of *H. influenzae* to levofloxacin was low, likely due to infrequent use of fluoroquinolones in children and newborns, as well as stringent regulations on quinolone antibiotic usage enforced by various departments in China. Over the period of 2017 to 2023, the resistance rate of *H. influenzae* to SXT ranged from 68.5% to 74.7%, SXT resistance in *H. influenzae* is usually caused by changes in the *folp* gene and the *sul2* gene [36], aligning with previous reports, suggesting that sulfonamides may no longer be suitable as empirical therapeutics for *H. influenzae* bloodstream infections. While the resistance rate to chloramphenicol (5.6%) was relatively low in this study, caution is advised in its clinical use due to significant adverse reactions in children.

## 5 Conclusion

The COVID‐19 pandemic has markedly influenced the epidemiological patterns of *H. influenzae* in Shandong. This impact not only underscores the profound repercussions of the pandemic on microbial ecology but also offers valuable insights and strategic preparations for a potential “X‐disease” pandemic on a global scale. By conducting precise data analysis, we aim to furnish clinicians with enhanced scientific and expert medication recommendations, refining anti‐infection treatment protocols to better address present and future health challenges. These efforts are geared towards making meaningful contributions to global public health security.

## Author Contributions

**Yanmeng Sun:** data curation, formal analysis, investigation, methodology, project administration, supervision, visualization, writing – original draft. **Mengyuan Wang:** data curation, formal analysis, investigation, methodology, methodology. **Zheng Li:** data curation, formal analysis, investigation, resources, supervision. **Sijin Man:** conceptualization, data curation, formal analysis, investigation. **Shifu Wang:** conceptualization, data curation, formal analysis, funding acquisition, investigation, methodology, project administration, resources, software, supervision, validation, visualization, writing – review and editing.

## 1 Conflicts of Interest

The authors declare no conflicts of interest.

## Transparency Statement

The lead author Shifu Wang affirms that this manuscript is an honest, accurate, and transparent account of the study being reported; that no important aspects of the study have been omitted; and that any discrepancies from the study as planned (and, if relevant, registered) have been explained.

## Data Availability

The data that support the findings of this study are available from the corresponding author upon reasonable request.

## References

[1] F. Shooraj , B. Mirzaei , S. F. Mousavi , and F. Hosseini , “Clonal Diversity of *Haemophilus influenzae* Carriage Isolated From Under the Age of 6 Years Children,” BMC Research Notes 12, no. 1 (2019): 565. 31506105 10.1186/s13104-019-4603-7 PMC6737650

[2] R. S. W. Tsang and M. Ulanova , “The Changing Epidemiology of Invasive *Haemophilus influenzae* Disease: Emergence and Global Presence of Serotype a Strains That May Require a New Vaccine for Control,” Vaccine 35, no. 33 (2017): 4270–4275. 28666758 10.1016/j.vaccine.2017.06.001

[3] I. Ayouni , J. Maatoug , W. Dhouib , et al., “Effective Public Health Measures to Mitigate the Spread of COVID‐19: A Systematic Review,” BMC Public Health 21, no. 1 (2021): 1015. 34051769 10.1186/s12889-021-11111-1 PMC8164261

[4] O. Gasch , L. Badia‐Cebada , J. Carmezim , et al., “Effects of the COVID‐19 Pandemic on Incidence and Epidemiology of Catheter‐Related Bacteremia, Spain,” Emerging Infectious Diseases 28, no. 11 (2022): 2181–2189. 36191608 10.3201/eid2811.220547 PMC9622263

[5] CLSI , *Performance Standards for Antimicrobial Susceptibility Testing*, 33rd ed. CLSI supplement M100. Clinical and Laboratory Standards Institute. 2023.

[6] I. Fadhil , R. Soliman , S. Jaffar , et al., “Estimated Incidence, Prevalence, Mortality, and Registration of Childhood Cancer (Ages 0–14 Years) in the WHO Eastern Mediterranean Region: An Analysis of GLOBOCAN 2020 Data,” Lancet Child & Adolescent Health 6, no. 7 (2022): 466–473. 35605628 10.1016/S2352-4642(22)00122-5

[7] Q. Meng , W. Li , H. Jiang , et al., “Comparison of the Distribution and Changes in the Antibiotic Resistance of Clinical Bacterial Isolates From the Lower Respiratory Tract of Children in Shenzhen Before the Epidemic, During the Epidemic, and During the Period of Normalized Prevention and Control of COVID‐19,” Infectious Diseases and Therapy 12, no. 2 (2023): 563–575. 36598677 10.1007/s40121-022-00751-4 PMC9812007

[8] R. E. Baker , S. W. Park , W. Yang , G. A. Vecchi , C. J. E. Metcalf , and B. T. Grenfell , “The Impact of COVID‐19 Nonpharmaceutical Interventions on the Future Dynamics of Endemic Infections,” Proceedings of the National Academy of Sciences 117, no. 48 (2020): 30547–30553. 10.1073/pnas.2013182117 PMC7720203 33168723

[9] A. Gastaldi , D. Dona , E. Barbieri , et al., “COVID‐19 Lesson for Respiratory Syncytial Virus (RSV): Hygiene Works,” Children (Basel) 8, no. 12 (2021): 1144. 34943339 10.3390/children8121144 PMC8700687

[10] S. G. Sullivan , S. Carlson , A. C. Cheng , et al., “Where Has All the Influenza Gone? The Impact of COVID‐19 on the Circulation of Influenza and Other Respiratory Viruses, Australia, March to September 2020[J],” Euro Surveillance: Bulletin Européen Sur Les Maladies Transmissibles = European Communicable Disease Bulletin 25, no. 47 (2020): 2001847. 33243355 10.2807/1560-7917.ES.2020.25.47.2001847 PMC7693168

[11] D. Shaw , R. Abad , Z. Amin‐Chowdhury , et al., “Trends in Invasive Bacterial Diseases During the First 2 Years of the COVID‐19 Pandemic: Analyses of Prospective Surveillance Data From 30 Countries and Territories in the IRIS Consortium,” Lancet Digital Health 5, no. 9 (2023): e582–e593. 37516557 10.1016/S2589-7500(23)00108-5 PMC10914672

[12] P. Y. Su , A. H. Huang , C. H. Lai , H. F. Lin , T. M. Lin , and C. H. Ho , “Extensively Drug‐Resistant *Haemophilus Influenzae*—Emergence, Epidemiology, Risk Factors, and Regimen,” BMC Microbiology 20, no. 1 (2020): 102. 32345232 10.1186/s12866-020-01785-9 PMC7189504

[13] D. K. J. Pieren , M. C. Boer , and J. de Wit , “The Adaptive Immune System in Early Life: The Shift Makes It Count,” Frontiers in Immunology 13 (2022): 1031924. 36466865 10.3389/fimmu.2022.1031924 PMC9712958

[14] K. E. Fleming‐Dutra , A. L. Hersh , D. J. Shapiro , et al., “Prevalence of Inappropriate Antibiotic Prescriptions Among US Ambulatory Care Visits, 2010‐2011,” Journal of the American Medical Association 315, no. 17 (2016): 1864–1873. 27139059 10.1001/jama.2016.4151

[15] F. Xue , B. Xu , A. Shen , and K. Shen , “Antibiotic Prescriptions for Children Younger Than 5 Years With Acute Upper Respiratory Infections in China: A Retrospective Nationwide Claims Database Study,” BMC Infectious Diseases 21, no. 1 (2021): 339. 33845771 10.1186/s12879-021-05997-w PMC8040226

[16] S. Reardon , “Infections Reveal Inequality Between the Sexes,” Nature 534, no. 7608 (2016): 447. 27337319 10.1038/534447a

[17] R. Y. Chen , Y. F. Li , W. Long , et al., “[Survey on Tobacco Use and Associated Factors in Population in Shandong Province, 2016‐2017],” Zhonghua Liu Xing Bing Xue Za Zhi = Zhonghua Liuxingbingxue Zazhi 42, no. 7 (2021): 1200–1204. 34814531 10.3760/cma.j.cn112338-20200903-01123

[18] H. J. Wang , C. Q. Wang , C. Z. Hua , et al., “Antibiotic Resistance Profiles of *Haemophilus Influenzae* Isolates From Children in 2016: A Multicenter Study in China,” Canadian Journal of Infectious Diseases & Medical Microbiology = Journal Canadien Des Maladies Infectieuses Et De La Microbiologie Medicale/AMMI Canada 2019 (2019): 6456321. 10.1155/2019/6456321 PMC6710757 31485283

[19] C. Ma , Y. Zhang , and H. Wang , “Characteristics of *Haemophilus influenzae* Carriage Among Healthy Children in China: A Meta‐Analysis,” Medicine 102, no. 44 (2023): e35313. 37933036 10.1097/MD.0000000000035313 PMC10627696

[20] Q. Fernandes , V. P. Inchakalody , M. Merhi , et al., “Emerging COVID‐19 Variants and Their Impact on SARS‐CoV‐2 Diagnosis, Therapeutics and Vaccines,” Annals of Medicine 54, no. 1 (2022): 524–540. 35132910 10.1080/07853890.2022.2031274 PMC8843115

[21] L. Pandolfi , T. Fossali , V. Frangipane , et al., “Broncho‐Alveolar Inflammation in COVID‐19 Patients: A Correlation With Clinical Outcome,” BMC Pulmonary Medicine 20, no. 1 (2020): 301. 33198751 10.1186/s12890-020-01343-z PMC7668012

[22] L. Sun , Y. Jiang , H. Gao , et al., “Patterns of Pediatric and Adolescent Gynecologic Problems in China: A Hospital‐Based Retrospective Study of 97,252 Patients,” Journal of Pediatric and Adolescent Gynecology 35, no. 4 (2022): 444–449. 35143978 10.1016/j.jpag.2022.01.010

[23] M. Dei , F. Di Maggio , G. Di Paolo , and V. Bruni , “Vulvovaginitis in Childhood,” Best Practice & Research Clinical Obstetrics & Gynaecology 24, no. 2 (2010): 129–137. 19884044 10.1016/j.bpobgyn.2009.09.010

[24] S. Baka , S. Demeridou , G. Kaparos , et al., “Microbiological Findings in Prepubertal and Pubertal Girls With Vulvovaginitis,” European Journal of Pediatrics 181, no. 12 (2022): 4149–4155. 36163515 10.1007/s00431-022-04631-4 PMC9649474

[25] X. Chen , L. Chen , W. Zeng , and X. Zhao , “ *Haemophilus influenzae* vulvovaginitis Associated With Rhinitis Caused by the Same Clone in a Prepubertal Girl,” Journal of Obstetrics and Gynaecology Research 43, no. 6 (2017): 1080–1083. 28621044 10.1111/jog.13311

[26] S. Wen , D. Feng , D. Chen , et al., “Molecular Epidemiology and Evolution of *Haemophilus influenzae* ,” Infection, Genetics and Evolution 80 (2020): 104205. 10.1016/j.meegid.2020.104205 31981610

[27] E. Heinz , “The Return of Pfeiffer's Bacillus: Rising Incidence of Ampicillin Resistance in *Haemophilus influenzae* ,” Microbial Genomics 4, no. 9 (2018): e000214. 30207515 10.1099/mgen.0.000214 PMC6202453

[28] L. Ai , L. Fang , C. Zhou , B. Liu , Q. Yang , and F. Gong , “The Impact of the COVID‐19 Pandemic on *Staphylococcus aureus* Infections in Pediatric Patients Admitted With Community Acquired Pneumonia,” Scientific Reports 14, no. 1 (2024): 12737. 38977804 10.1038/s41598-024-66071-4 PMC11231152

[29] P. Fu , H. Xu , C. Jing , et al., “Bacterial Epidemiology and Antimicrobial Resistance Profiles in Children Reported by the ISPED Program in China, 2016 to 2020,” Microbiology Spectrum 9, no. 3 (2021): e0028321. 34730410 10.1128/Spectrum.00283-21 PMC8567242

[30] S. Bae , J. Lee , J. Lee , et al., “Antimicrobial Resistance in *Haemophilus influenzae* Respiratory Tract Isolates in Korea: Results of a Nationwide Acute Respiratory Infections Surveillance,” Antimicrobial Agents and Chemotherapy 54, no. 1 (2010): 65–71. 19884366 10.1128/AAC.00966-09 PMC2798543

[31] Z. Assad , R. Cohen , E. Varon , et al., “Antibiotic Resistance of *Haemophilus influenzae* in Nasopharyngeal Carriage of Children With Acute Otitis Media and in Middle Ear Fluid From Otorrhea,” Antibiotics (Basel) 12, no. 11 (2023): 1605. 37998807 10.3390/antibiotics12111605 PMC10668799

[32] V. C. Cheng , S. C. Wong , S. Y. So , et al., “Decreased Antibiotic Consumption Coincided With Reduction in Bacteremia Caused by Bacterial Species With Respiratory Transmission Potential During the COVID‐19 Pandemic[J],” Antibiotics (Basel) 11, no. 6 (2022): 746. 35740153 10.3390/antibiotics11060746 PMC9219721

[33] S. Yamada , S. Seyama , T. Wajima , et al., “β‐Lactamase‐non‐producing Ampicillin‐Resistant *Haemophilus influenzae* Is Acquiring Multidrug Resistance,” Journal of Infection and Public Health 13, no. 4 (2020): 497–501. 31839585 10.1016/j.jiph.2019.11.003

[34] X. Zhong , A. Pate , Y. T. Yang , et al., “Impact of COVID‐19 on Broad‐Spectrum Antibiotic Prescribing for Common Infections in Primary Care in England: A Time‐Series Analyses Using OpenSAFELY and Effects of Predictors Including Deprivation,” Lancet Regional Health—Europe 30 (2023): 100653. 37363797 10.1016/j.lanepe.2023.100653 PMC10186397

[35] GBD 2021 Lower Respiratory Infections and Antimicrobial Resistance Collaborators, “ Infections GBDLR, Antimicrobial Resistance C. Global, Regional, and National Incidence and Mortality Burden of Non‐COVID‐19 Lower Respiratory Infections and Aetiologies, 1990–2021: A Systematic Analysis From the Global Burden of Disease Study 2021,” Lancet Infectious Diseases 24, no. 9 (2024): 974–1002. 38636536 10.1016/S1473-3099(24)00176-2 PMC11339187

[36] T. W. Wan , Y. T. Huang , J. H. Lai , et al., “The Emergence of Transposon‐Driven Multidrug Resistance in Invasive Nontypeable *Haemophilus influenzae* Over the Last Decade,” International Journal of Antimicrobial Agents 64, no. 4 (2024): 107319. 39233216 10.1016/j.ijantimicag.2024.107319

